# Finite element analysis of the proximal femoral growth plate biomechanics: insights into cam deformity risk of adolescent athletes

**DOI:** 10.3389/fspor.2026.1746084

**Published:** 2026-07-14

**Authors:** Yifan Jiang, Zhexiong Tang, Xiaoyuan Gong, Tiao Su, Guangxing Chen

**Affiliations:** 1 Center for Joint Surgery, Intelligent Manufacturing and Rehabilitation Engineering Center, The First Hospital Affiliated to Army Medical University, Chongqing, China; 2 Chongqing Municipal Science and Technology Bureau Key Laboratory of Precision Medicine in Joint Surgery, Chongqing, China; 3 Chongqing Municipal Education Commission Key Laboratory of Joint Biology, Chongqing, China; 4 Sanya Rehabilitation and Convalescent Center, Joint Logistics Support Force, Sanya, China

**Keywords:** biomechanical influences, cam deformity, epiphyseal growth plate, proximal femur, stress distribution

## Abstract

**Objectives:**

To investigate the biomechanical influences on the morphology and development of the proximal femur by analyzing the stress distribution within the proximal femoral epiphyseal growth plate (EGP) of adolescents during both daily activities and sports.

**Methods:**

Three-dimensional pelvic CT data from adolescents were employed to construct a finite element mechanical analysis (FEMA) model of the hip joint. Real-time, continuous data on hip forces and angles were collected during daily activities and sports using a gait and joint mechanics acquisition system. Representative mechanical loads of the femur and hip joint were selected from the collected data as loading conditions. A three-dimensional FEMA of the hip joint under these conditions was performed to evaluate stress distribution across the growth plate and its nine distinct zones.

**Results:**

Compared with daily activities, basketball dribbling and layups, as well as football shooting and passing, resulted in higher concentrations of compressive stress within the lateral region of the growth plate, whereas ice hockey demonstrated greater compressive stress in the central regions, extending laterally. Basketball layups exhibited concentrated shear stress within the medial and central regions. Football dribbling and passing showed increased shear stress in the medial regions, while ice hockey exhibited a concentration of shear stress in the medial regions.

**Conclusion:**

High-impact sports produce concentrated lateral compressive stress and medial shear stress on the growth plate, forming mechanical stimuli distinct from daily activities. These differential stress distributions provide a reference for exploring mechanical features associated with adolescent hip morphological variation.

## 1 Introduction

Femoroacetabular impingement (FAI) is a significant contributor to hip pain in young adults, typically presenting in two forms: cam and pincer, with mixed morphologies also frequently observed (1–3). Among these, cam deformity is more prevalent and is regarded as a precursor to osteoarthritis (OA), making it a prominent focus of current research (4–9). At present, no universally standardized protocol exists for diagnosing or treating FAI. Surgical options, ranging from joint-preserving orthopedic procedures to hip arthroplasty, demonstrate good short- and medium-term efficacy and may lower the incidence of osteoarthritis; however, their ability to fully prevent osteoarthritis remains unproven (4, 5, 10). Therefore, for FAI, a developmental abnormality in bony structures, it is crucial to investigate its etiology and identify the key mechanisms that trigger abnormal morphological development of hip bone tissue.

Research has indicated that factors such as sex, genetics, trauma, and physical activity may all play a role in the development of cam deformity (11). Notably, athletes involved in activities imposing high-impact loads on the hip joint exhibit a prevalence of cam deformity as high as 89%, compared with only 9% in non-athlete controls (1, 2, 12, 13). This stark contrast suggests that high-intensity sports may significantly contribute to the onset and progression of cam deformity during adolescence.

The growth plate, or epiphyseal plate, is a crucial structure for the longitudinal growth of long bones and vertebral bodies, achieved through continuous calcification of newly formed cartilage (14, 15). Mechanical loads are recognized as key regulators of longitudinal bone growth (16–18). According to Hueter-Volkmann's law, excessive compressive stress inhibits bone growth, whereas tensile or shear stresses within physiological limits (without causing structural damage) stimulate bone growth (19, 20). According to Hueter-Volkmann's law, we hypothesize that prolonged training in sports such as basketball, football, and ice hockey, particularly involving high-impact actions such as layups and shooting, may lead to increased lateral compressive stress, thereby slowing the growth rate of the lateral growth plate, while concentrated medial shear stress could relatively promote vertical growth of the medial growth plate. Therefore, understanding the stress distribution within the proximal femoral growth plate during various daily activities and sports is crucial for elucidating the biomechanical mechanisms that underpin the development of abnormal proximal femoral morphology.

Finite element analysis (FEA) is widely applied across musculoskeletal biomechanics beyond hip research, as exemplified by Zhou et al. (21), who adopted FE simulation to explore foot biomechanical changes following fifth metatarsal fracture during running. Despite substantial progress, current FEA research on proximal femoral growth plates faces two primary limitations. Firstly, finite element models often lack sufficient anatomical accuracy to replicate the true morphology of the growth plate. Secondly, the mechanical loading conditions used do not adequately reflect the dynamic environment experienced by the femoral head during physical activity. Studies by Yadav et al., Castro et al., and Kandzierski et al. (22–25) reconstructed proximal femoral growth plates of children aged 7–11 years using computed tomography (CT) and magnetic resonance imaging (MRI) data to analyse geometric differences in growth plate models. However, their simplified layered models did not achieve a biomimetic representation of the proximal femoral growth plate. Moreover, current studies on hip joint loading conditions predominantly refer to the work of Bergmann et al. (26–28), which employs fixed mechanical load models that fail to capture the dynamic nature of loads experienced by the hip joint during movement.

The present study aims to develop a biomimetic three-dimensional (3D) finite element model (FEM) of the proximal femoral epiphyseal growth plate (EGP). By establishing a comprehensive database of mechanical loads across various activity modes, this study seeks to capture representative loading conditions during both daily activities and diverse sports. This approach aims to elucidate the variations in mechanical distribution under different conditions and their impact on growth plate elongation, ultimately laying the foundation for a deeper understanding of the mechanical mechanisms underlying the formation of cam deformity.

## 2 Materials and methods

### 2.1 Finite element model construction

Pelvic data (CT slice thickness: 1.0 mm, 120 kV, 3 mAS; GE, USA) were utilised for constructing the model, excluding cases involving hip deformities, trauma, or extension. Ethical approval for the study was granted by the Ethics Committee of our hospital (No. KY2023065).

**Figure 1:**
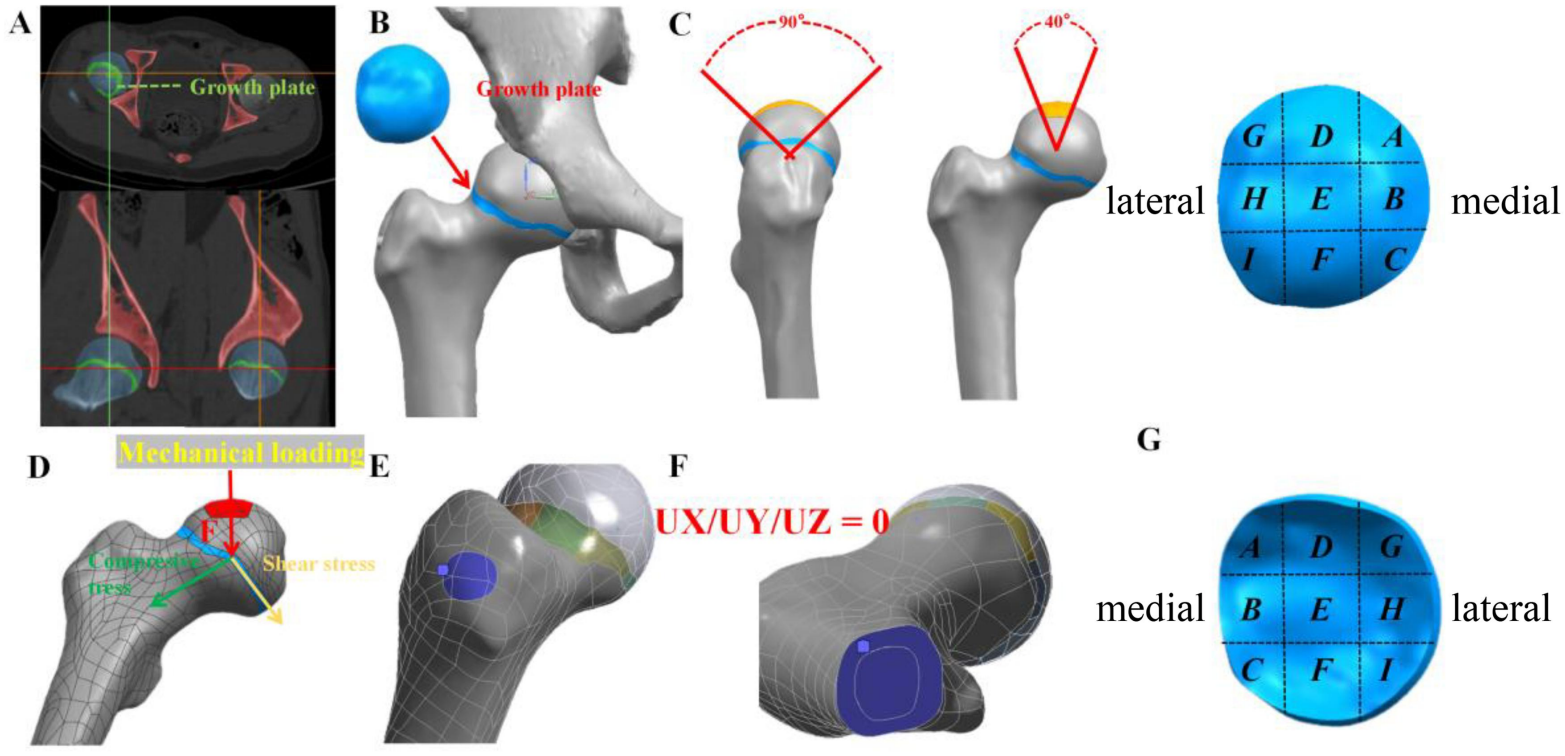
Comprehensive analysis of the growth plate and loading conditions. **(A)** Reconstruction of the femur, femoral head, and growth plate from CT data; **(B)** Reconstruction of the sacroiliac joint and femoral head, yielding the growth plate model; **(C)** Identification of the load application area; **(D)** Specification of loading direction, conditions, and methodology; **(E)** Application of mechanical constraints at the greater trochanter; **(F)** Application of mechanical constraints at the distal femur; **(G)** Zonal segmentation model of the EGP for detailed analysis.

CT data from a 14-year-old male subject were used to construct a hip joint model with a normal growth plate, with images clearly showing an unclosed epiphysis (Figure 1A). Using Mimics 21.0 (Materialise), 3D models of the femur, pelvis, and growth plate were reconstructed, followed by conversion into surface models using Geomagic Wrap (Raindrop). Subsequently, these surface models were assembled with Siemens NX12.0 software (Siemens) to create models representing different hip joint states during physical activity.

The hip joint finite element model was established in ANSYS Workbench 2021. Isotropic linear elastic material properties were assigned to distinct tissues: the growth plate cartilage possessed an elastic modulus of 5 MPa and a Poisson's ratio of 0.48; cancellous bone was set to 445 MPa with a Poisson's ratio of 0.22; cortical bone adopted an elastic modulus of 15.1 GPa and a Poisson's ratio of 0.3.

All anatomical domains were discretized using 10-node quadratic tetrahedral SOLID187 elements. A refined mesh size of 0.5 mm was applied to the growth plate to capture subtle intra-cartilage stress gradients, while all bony structures were meshed uniformly at 1.0 mm. Detailed mesh statistics including element counts and node quantities for each tissue compartment are documented in Supplementary Table S1.

Rigorous global mesh quality criteria were implemented across the entire model: the maximum element aspect ratio was limited below 5, element skewness was controlled under 0.85, and the Jacobian ratio was maintained above 0.5. To accurately resolve stress variations through cartilage thickness, a minimum of 3–4 element layers were distributed across the full depth of the growth plate.

A mesh convergence study was exclusively conducted on the growth plate to confirm mesh-independent stress predictions, with results for four tested grid resolutions compiled in Supplementary Table S2. The relative deviation in peak stress between the 1 mm and 0.5 mm discretization reached merely 2.23%, satisfying the widely accepted 5% convergence criterion. Further refining the mesh from 0.75 mm to 0.5 mm only reduced peak stress by a trivial 1.95%, yet computational runtime increased by 2.26 times. Balancing numerical precision and computational cost, we ultimately adopted the 0.5 mm mesh for the growth plate and 1.0 mm meshes for bone tissues. All elements were inspected to eliminate severely distorted geometries and guarantee stable numerical computation.

### 2.2 Collection of loading conditions

Five healthy male volunteers with athletic proficiency (mean height: 167.8 ± 2.95 cm, mean weight: 65.3 ± 3.42 kg) were recruited. Participants with a history of lower limb disorders or surgeries were excluded. Volunteers underwent standardised training for daily activities (e.g., walking, single-leg/double-leg standing, sitting and standing, squatting, stair ascent/descent) and sport-specific activities (e.g., basketball layups, dribbling, shooting; football dribbling, passing, shooting; ice hockey shooting, dribbling, sharp turns).

**Figure 2:**
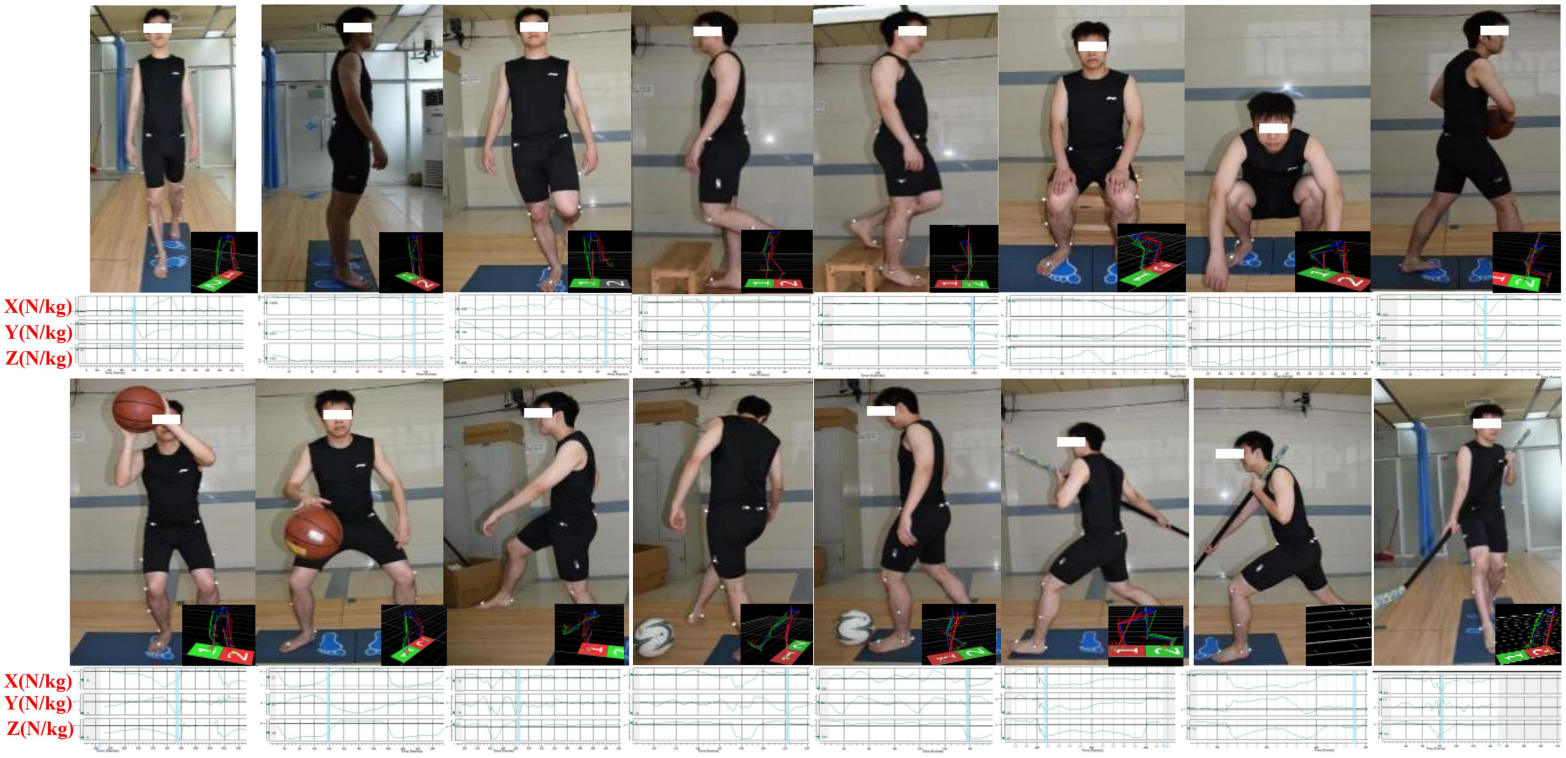
Volunteers performing pre-defined daily and sports activities. Top row, left to right: walking, double-leg standing, single-leg standing, ascending stairs, descending stairs, sitting and standing, squatting and standing, basketball layup; bottom row, left to right: two-handed shooting, basketball dribbling, football shooting, passing, dribbling, ice hockey shooting, dribbling, and turning.

A Vicon optical capture system (Oxford Metrics Limited, UK) with 12 cameras was used to dynamically capture (sampling frequency was set to 100 Hz) the motion trajectories (joint angles, angular velocity, angular acceleration) of these activities and an AMTI 3D force plates (AMTI, USA) was applied to collect plantar forces. The hip joint contact force was calculated by using the Plug-in Gait Kinematic and kinetic calculation module in Vicon system through plantar stress. Before the official collection, volunteers were already proficient in completing various actions at the collection site and meeting the collection requirements. Each volunteer performed each movement five times (29–31), with hip joint angles and force measurements recorded at three key statuses: the start, end, and peak stress (Figure 2). Using the methodology of Bergmann et al. (27), mean values for significant mechanical data points, including movement onset, completion, and peak values, were selected as loading conditions for the finite element model analysis (Supplementary Table S1).

### 2.3 Finite element analysis loading approach

Loading was applied in a surface manner, as described in previous literature (24, 32–36). The force application area on the femoral head was defined as follows: the center of rotation was angled 45° forward and backward (90° arc of the sphere), and 20° medially, simulating the load-bearing region from the acetabulum apex to its lateral boundary (Figure 1C). The direction of loading was defined according to the spatial orientation of the hip joint (Table 1; Figure 1D). The lower surface of the distal femur and the region around the greater trochanter were fully fixed, with all three translational nodal displacements (UX, UY, UZ) constrained to zero (Figures 1E,F).

**Table 1:** Mesh convergence study.

Grid size (mm)	Number of nodes	peak stress (MPa) vs. 1 mm mesh (%)	Calculations take time (min)
2	2,69,676	0.7034 (−23.43)	84
1	3,43,850	0.9186 (0.00)	222
0.75	4,52,848	0.9578 (+4.30)	1,044
0.5	8,84,726	0.9,391 (+2.23)	2,351

**Figure 3:**
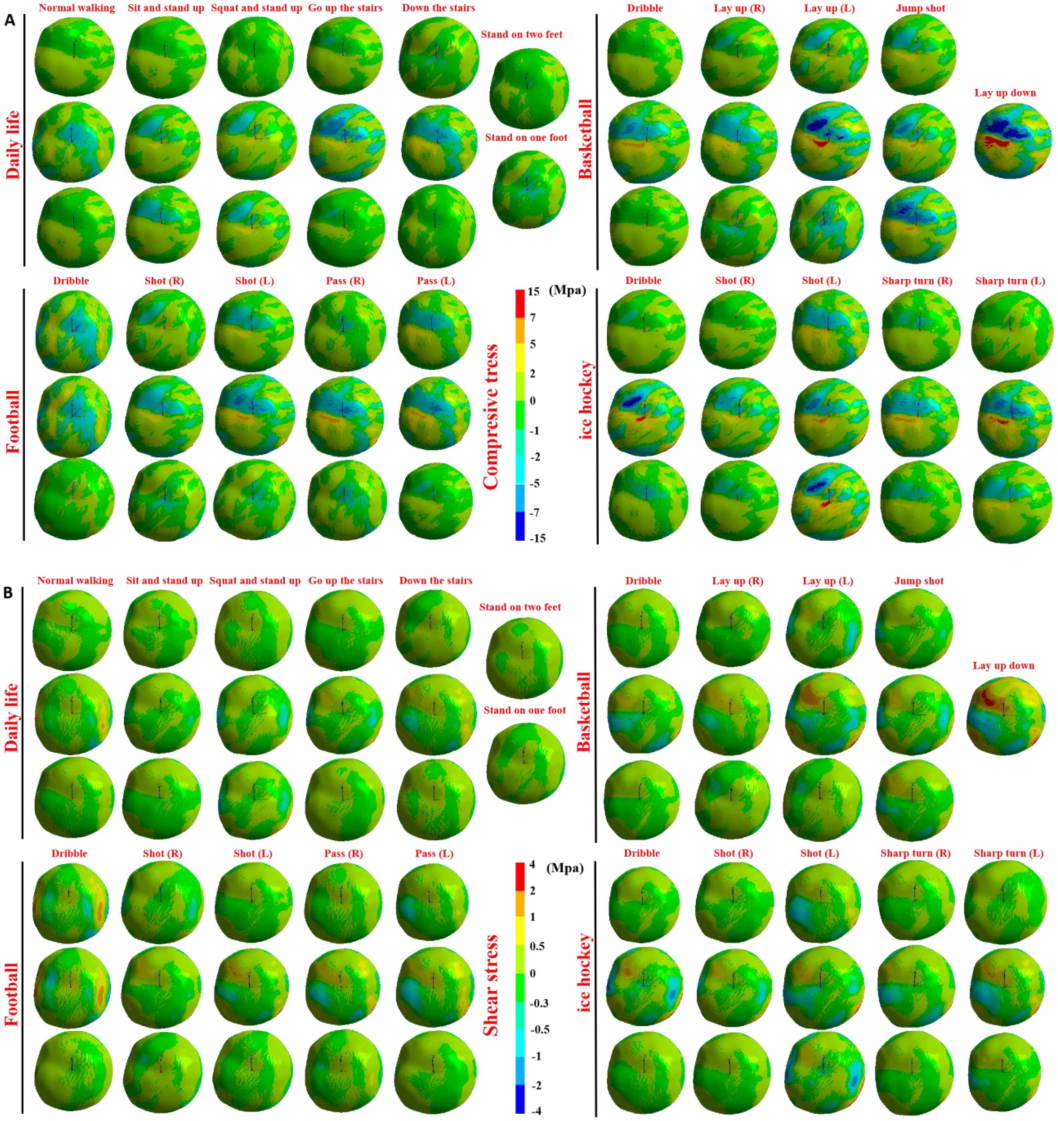
Stress distribution on the growth plate during daily and sports activities. Each row corresponds to three key statuses of one movement type.**(A)** Compressive stress distribution on the growth plate during both daily and sports activities; **(B)** Shear stress distribution on the growth plate during both daily and sports activities.

The FEA model was assembled in ANSYS Workbench 2021. To facilitate localized stress evaluation, the growth plate was segmented into nine analytical subzones (A–I; Figure 1G). Subdivision was conducted on the top-down planar view of the complete growth plate surface. The full physeal area was equally divided into three segments along the mediolateral width and three segments along the anteroposterior length, forming a standardized 3 × 3 grid comprising nine independent subzones. Typical mechanical loading conditions during daily activities and sports were applied to observe the resultant stress distribution across the entire growth plate and within each of the nine zones. After finite element calculation, 120 contour plots of compressive stress and shear stress were generated for post-processing (Figure 3). Through overall evaluation and analysis of all contour plots, a total of 1,080 valid stress values were extracted to form the final analysis dataset for subsequent quantitative comparison.

### 2.4 Statistical analysis

Hip angles and forces were summarized as arithmetic means, while peak stress values in the growth plate were reported as mean ± standard deviation. Heatmaps were applied to characterize spatial stress distribution among anatomical subzones, and violin plots were used to show the overall distribution of stress magnitudes for four categories of movement conditions (daily activities, basketball, football, ice hockey). The statistical methodology followed the CHAMP guidelines, as recommended by Mansournia et al. (37).

## 3 Results

### 3.1 Distribution and magnitude of compressive stress in the EGP

#### 3.1.1 Overall trends

**Figure 4:**
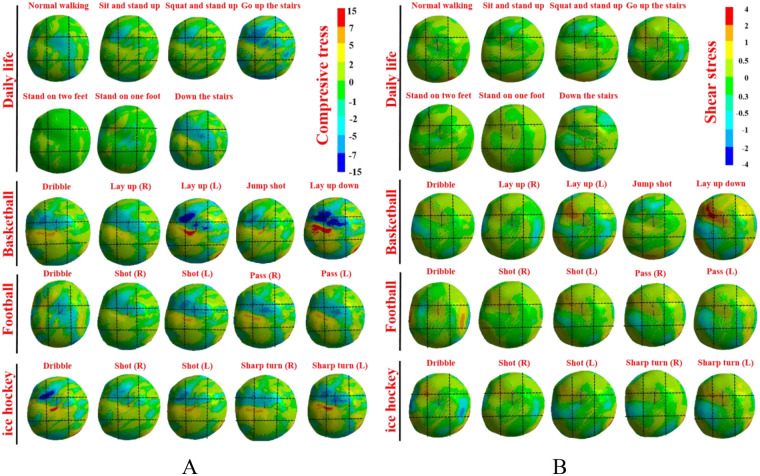
Peak stress distribution on the growth plate during daily and sports activities. **(A)** Peak compressive stress distribution on the growth plate during daily and sports activities; **(B)** Peak shear stress distribution on the growth plate during daily and sports activities.

**Figure 5:**
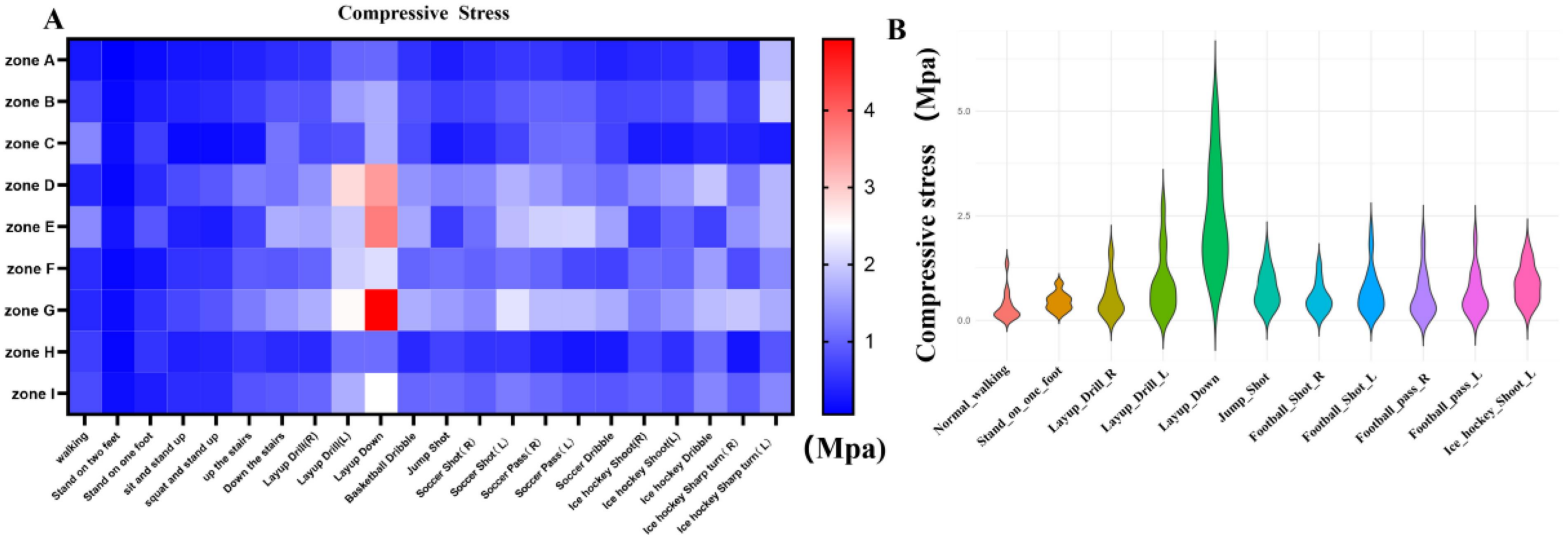
Comparative analysis of compressive stress during daily and sports activities. **(A)** Compressive stress comparison of nine subzones between daily and sports; **(B)** Top ten activities showing the greatest differences in compressive stress compared with walking.

For compressive stress results, positive values indicate tensile stress and negative values indicate compressive stress. In the context of normal daily activities, no significant variations in the distribution of compressive stress were observed across different regions of the EGP. However, during sports activities, compared with normal daily activities, basketball actions such as dribbling and layups, as well as football maneuvers like shooting and passing, exhibited more localized compressive stress concentrated in the lateral H and E regions. Conversely, ice hockey demonstrated a greater concentration of compressive stress within central regions extending laterally (Figure 4A). When comparing average compressive stress, both football and basketball activities showed heightened stress in the medial zones, with ice hockey demonstrating additional concentrated compressive stress in the lateral G region (Figure 5A).

#### 3.1.2 Peak compressive stress in daily activities

Among daily activities, walking exhibited the highest peak compressive stress of 7.79 MPa, although it did not significantly differ from the compressive stresses observed during stair ascent and descent, which also demonstrated elevated values. During the initiation and termination phases of walking, compressive stresses were substantially lower, similar to those recorded during double-leg standing. Double-leg standing yielded the lowest peak compressive stress of 1.86 MPa, which was significantly lower than all other activities. Although single-leg standing induced slightly higher compressive stresses than double-leg standing, it remained substantially lower than walking, sitting down and rising, or descending stairs.

#### 3.1.3 Peak compressive stress in sports activities

The highest peak compressive stress recorded was during basketball layup landings at 28.65 MPa, which was significantly greater than those in all other sports activities. The lowest peak compressive stress during sports was associated with football passing (L), with a value of 6.49 MPa. Moreover, explosive movements, including jumping, shooting, and sharp turns across all three sports, generally produced elevated peak compressive stresses compared to other actions.

Excluding walking, peak compressive stress values during normal daily activities were markedly lower compared to those in sports activities (Figure 5B).

### 3.2 Distribution and magnitude of shear stress in the EGP

#### 3.2.1 Overall trends

**Figure 6:**
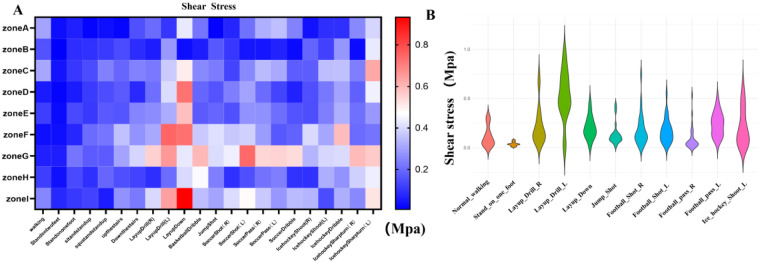
Comparative analysis of shear stress during daily and sports activities. **(A)** Shear stress comparison of nine subzones between daily and sports; **(B)** Top ten activities demonstrating the greatest differences in shear stress compared with walking.

For shear stress in this model, positive values refer to shear causing clockwise rotation of EGP, and negative values refer to shear causing counterclockwise rotation. During routine daily activities, no significant differences were detected in the distribution of shear stress among the different regions of the EGP. However, during sports activities, basketball layups induced elevated shear stress concentrations in the medial (A, C), posterior (D, G), and central (E) regions. Football dribbling and passing exhibited increased shear stress in the medial (A, B, C) regions, whereas ice hockey induced concentrated shear stress in the medial (B, C) regions (Figure 4B). Comparatively, basketball and football showed higher average shear stress concentrations in the A region (Figure 6A).

#### 3.2.2 Peak shear stress in daily activities

Within normal daily activities, double-leg standing resulted in significantly lower peak shear stress than all other activities, with the lowest value at 0.85 MPa. Single-leg standing yielded slightly higher shear stress compared to double-leg standing but remained significantly lower than walking, sitting down and rising, or ascending and descending stairs. Walking exhibited the highest peak shear stress at 4.57 MPa; however, the differences in shear stress among walking, sitting down and rising, squatting, and stair climbing were not statistically significant.

#### 3.2.3 Peak shear stress in sports activities

Basketball layup landings produced the highest peak shear stress among all sporting activities, reaching 11.91 MPa, which was significantly greater than that observed during other sporting actions. The lowest peak shear stress during sports was associated with football dribbling, at 4.28 MPa. Except for football shooting, explosive actions, such as jumping and shooting across the three sports, consistently resulted in higher peak shear stresses compared to other actions.

With the exception of football dribbling and ice hockey sharp turns (R), the peak shear stress values observed during various sporting activities were notably higher than those during daily activities (Figure 6B).

## 4 Discussion

This study demonstrated that sports activities impose significantly higher compressive and shear stresses on the proximal femoral EGP compared to normal daily activities. Notably, basketball layups generated the highest peak compressive (28.65 MPa) and shear stresses (11.91 MPa), indicating that explosive sports actions markedly increase mechanical loading on the EGP. Regional zonal analysis illustrated evident heterogeneous stress distribution patterns: compressive stress tended to concentrate in lateral EGP zones, while shear stress was mainly distributed in medial and central EGP regions during sports movements. Collectively, these results confirm that high-impact sports trigger elevated, uneven and regionally localized mechanical stimuli across the growth plate, which may disturb physiological EGP growth and further facilitate the occurrence of cam-type FAI. To better interpret these mechanical findings, we combine well-recognized mechanobiological theories and unresolved research gaps in current literature.

Although clinical relevance between abnormal EGP elongation and subsequent FAI has been well documented in previous epidemiological and imaging studies (1, 2, 13, 38–40), the specific coupled biomechanical pathways accounting for sports-induced EGP morphological changes remain unclarified. Existing clinical evidence (1, 2, 21, 23) has confirmed a huge disparity in cam deformity prevalence: athletes receiving chronic high hip impact loads present a cam deformity rate up to 89%, whereas the prevalence is only 9% among matched non-athletic adolescents. Nevertheless, these clinical observations only confirm population-level correlation rather than revealing internal mechanical causes. Classic mechanobiology theories (20, 41–43) have illustrated the regulatory effects of mechanical stimuli on endochondral ossification and bone growth. Consistent with Carter's mechanical regulation theory (44, 45), sustained excessive compressive stress inhibits cartilage ossification, while positive shear stress produces clockwise rotation of the EGP, whereas negative shear stress induces counterclockwise rotation. Opposite rotational shear loads apply divergent tangential forces across layered physeal cartilage. Persistent clockwise or counterclockwise shear disrupts uniform chondrocyte column arrangement and may modulate regional chondrocyte proliferation, creating uneven mechanical signals that potentially affect localized growth rates. Built on these recognized theories and clinical phenomena, our finite element results further fill the mechanical mechanism gap: our quantified asymmetric regional stress data directly explains how differentiated compressive and shear stress distribution disrupts synchronized EGP growth, connecting macroscopic clinical deformity phenomena with microscopic biomechanical stress characteristics.

Previous hip finite element simulations have reconstructed proximal femoral models based on clinical CT or MRI scans with realistic overall femoral and pelvic geometry, yet most of these studies adopted a simplified layered modelling approach for the epiphyseal growth plate (21, 24, 35, 46). Specifically, they divided the entire growth plate into several uniform, parallel flat layers with consistent thickness and regular geometric shapes, ignoring the natural irregular curvature, uneven thickness and undulating three-dimensional anatomical morphology of native adolescent EGP. Such oversimplified layered structures cannot replicate the authentic anatomical characteristics of physiological growth plates, inevitably introducing calculation errors and reducing the credibility of simulated stress outcomes. In contrast, the term biomimetic model used in our study refers to a patient-specific solid growth plate model reconstructed strictly following the real anatomical contour, variable thickness and irregular curved morphology of the 14-year-old adolescent's native EGP without manual geometric simplification. This biomimetic modelling strategy retains the original complex spatial structure of the growth plate, which is much closer to the *in-vivo*anatomical state and effectively improves the accuracy of subsequent biomechanical stress analysis.

Most studies examining the loading conditions of hip joint mechanics refer to the seminal work by Bergmann et al. (25–27), which primarily investigated femoral head mechanical loading during daily activities. However, previous research (11, 32, 47–51) has shown that the incidence of FAI is markedly higher among athletes engaged in sports such as basketball, football, and ice hockey compared to the general population. Despite this, detailed data regarding the mechanical loads on the hip joint during these sports are lacking. Additionally, existing mechanical loading patterns (e.g., fixed forces and directions) do not adequately reflect the true dynamic loading conditions experienced during real motion, which compromises the accuracy of experimental findings. In this study, we employed a joint hip mechanics loading data acquisition system that incorporates gait analysis and plantar stress feedback to capture continuous movement details and dynamic mechanical data. The activity modes studied included not only daily activities but also various sports actions, such as basketball (layups, shooting, dribbling), football (shooting, dribbling, passing), and ice hockey (shooting, dribbling, sharp turns), thereby facilitating a comprehensive analysis of hip joint mechanical loads during different sports. Multiple mechanical loading modes, positions, and force magnitudes were applied to evaluate their effects on the growth plate, resulting in a more thorough and complete dataset, with more accurate and realistic stress distribution findings.

Building upon a normal growth plate model, previous studies (21, 24, 35, 52–55) have only examined the apparent mechanical distribution across the growth plate. In contrast, this study employed an innovative zonal method for growth plate analysis, wherein each zone was independently analyzed before integration to quantitatively evaluate stress distribution. Our results show that compressive and shear stress magnitudes under daily routines tend to be lower than those recorded during athletic movements. Static weight-bearing postures including single- and double-limb standing generally generate milder mechanical loads, which correspond to reduced potential mechanical stimuli on the growth plate.Within daily functional motions, similar compressive and shear stress ranges and distribution patterns were observed across walking, squat-to-stand transitions, sit-to-stand movements, and stair ascent and descent. Among athletic tasks, basketball layup landings and ice hockey shooting produced notably elevated peak compressive and shear loads relative to everyday activities and other sport-specific movements, which suggests these two motions may impose greater mechanical burden on the epiphyseal growth plate.

Further zonal analysis revealed that, compared with daily activities, compressive stress during basketball and football was predominantly localized in the lateral H and E regions of the EGP, while compressive stress in ice hockey concentrated in the central E region and extended toward the G region. These findings suggest that sports impose higher lateral compressive stresses than daily activities. Moreover, shear stress during basketball was more concentrated in the medial (A, C), posterior (D, G), and central (E) regions, while football exhibited greater shear stress in the medial (A, B, C) zones, and ice hockey showed more concentrated shear stress in the medial (B, C) zones. These observed asymmetric stress distributions are consistent with our initial research hypothesis. Combined with Hueter-Volkmann's law, we can elaborate the underlying biomechanical mechanism: prolonged engagement in high-impact actions such as layups and shooting in basketball, football and ice hockey keeps the lateral EGP under persistent high compressive stress and slows its growth. By contrast, long-term clockwise or counterclockwise rotational shear generates mismatched tangential mechanical cues between medial and lateral cartilage regions, which may create imbalanced growth regulation signals and continuous medial shear stress facilitates vertical growth of the medial growth plate. These asymmetric stress distributions align with our initial research hypothesis. Such unbalanced growth between the two sides leads to lateral extension of the EGP, which is a key precursor to cam deformity.

In summary, high-intensity sports activities result in increased and asymmetrical stress distribution in the proximal femoral growth plate of adolescents, potentially leading to lateral growth plate extension, thereby offering new insights into the underlying mechanisms of cam deformity formation at the femoral head-neck junction.

### 4.1 Limitations

Several limitations exist in this study. First, only instantaneous stress at discrete motion time points was calculated; the dose–response relationship between loading duration/magnitude and physeal dysplasia remains unquantified. Second, standardized lab movements cannot fully replicate irregular in-game athletic motions. Third, simulations relied on one representative adolescent FE model without accounting for inter-individual anatomical variation. Fourth, only healthy intact growth plates were analyzed, while pathological elongated physeal tissue was not evaluated.

Additionally, subject-specific muscle forces were excluded. Since muscle loads strongly modulate hip biomechanics, this simplification may shift predicted stress magnitudes and distributions, though the unified model still supports valid cross-movement comparisons. Kinematic data were collected from merely five male volunteers with similar body size, restricting the generalizability of loading conditions across adolescent athletes of diverse stature and skill levels. Follow-up cohort biomechanical studies will address these drawbacks.

## 5 Conclusions

Basketball, football and ice hockey induce concentrated compressive stress in the lateral growth plate and concentrated shear stress in the medial region, which differ from the stress profiles under routine daily movements. The varied regional stress characteristics induced by different loading conditions offer a biomechanical reference for analyzing mechanical environments around the adolescent proximal femoral growth plate.

## Data Availability

The original contributions presented in the study are included in the article/Supplementary Material, further inquiries can be directed to the corresponding authors.

## References

[1] Agricola R Heijboer MP Bierma-Zeinstra SM Verhaar JAN Weinans H Waarsing JH. Cam impingement causes osteoarthritis of the hip: a nationwide prospective cohort study (CHECK). Ann Rheum Dis. (2013) 72(6):918–23. 10.1136/annrheumdis-2012-201643 22730371

[2] Agricola R Waarsing J Arden N Carr AJ Bierma-Zeinstra SMA Thomas GE. Cam impingement of the hip: a risk factor for hip osteoarthritis. Nat Rev Rheumatol. (2013) 9(10):630–34. 10.1038/nrrheum.2013.114 23881070

[3] Kemp JL Makdissi M Schache AG Pritchard MG Pollard TCB Crossley KM. Hip chondropathy at arthroscopy: prevalence and relationship to labral pathology, femoroacetabular impingement and patient-reported outcomes. Br J Sports Med. (2014) 48(14):1102–07. 10.1136/bjsports-2013-093312 24659505

[4] Griffin DR Dickenson EJ Wall PDH Achana F Donovan JL Hobson R. Hip arthroscopy versus best conservative care for the treatment of femoroacetabular impingement syndrome (UK FASHIoN): a multicentre randomised controlled trial. Lancet. (2018) 391(10136):2225–35. 10.1016/S0140-6736(18)31202-9 29893223 PMC5988794

[5] Khan M Bedi A Fu F Karlsson J Ayeni OR Bhandari M. New perspectives on femoroacetabular impingement syndrome. Nat Rev Rheumatol. (2016) 12(5):303–10. 10.1038/nrrheum.2016.17 26963727

[6] Atkins PR Shin Y Agrawal P Elhabian SY Whitaker RT Weiss JA. Which two-dimensional radiographic measurements of cam femoroacetabular impingement best describe the three-dimensional shape of the proximal femur? Clin Orthop Relat R. (2019) 477(1):242–53. 10.1097/CORR.0000000000000462 PMC6345307 30179924

[7] Agricola R Weinans H. What causes cam deformity and femoroacetabular impingement: still too many questions to provide clear answers. Br J Sports Med. (2016) 50:263–64. 10.1136/bjsports-2015-094773 26486586

[8] Van Klij P Heerey J Waarsing JH Agricola R. The prevalence of cam and pincer morphology and its association with development of hip osteoarthritis. JOSPT. (2018) 48(4):230–38. 10.2519/jospt.2018.7816 29548271

[9] Nepple JJ. Editorial commentary: symptomatic femoroacetabular impingement resulting in severe hip osteoarthritis: predicting the “perfect storm”: arthroscopy. Arthroscopy. (2022) 38:1187–88. 10.1016/j.arthro.2021.11.001 35369920

[10] Dwyer T Whelan D Shah PS Ajrawat P Hoit G Chahal J. Operative versus nonoperative treatment of femoroacetabular impingement syndrome: a meta-analysis of short-term outcomes. Arthroscopy. (2020) 36(1):263–73. 10.1016/j.arthro.2019.07.025 31864588

[11] Nepple JJ Vigdorchik JM Clohisy JC. What is the association between sports participation and the development of proximal femoral cam deformity? A systematic review and meta-analysis. Am J Sports Med. (2015) 43(11):2833–40. 10.1177/0363546514563909 25587186

[12] Sankar WN Nevitt M Parvizi J Felson DT Agricola R Leunig M. Femoroacetabular impingement: defining the condition and its role in the pathophysiology of osteoarthritis. J Am Acad Orthop Surg. (2013) 21(Suppl 1):S7–S15. 10.5435/JAAOS-21-07-S7 23818194

[13] Siebenrock KA Ferner F Noble PC Santore RF Werlen S Mamisch TC. The cam-type deformity of the proximal femur arises in childhood in response to vigorous sporting activity. Clin Orthop Relat R. (2011) 469(11):3229–40. 10.1007/s11999-011-1945-4 PMC3183218 21761254

[14] Ballock RT O'Keefe RJ. The biology of the growth plate. J Bone Joint Surg Br. (2003) 85(4):715–26. 10.2106/00004623-200304000-00021 12672851

[15] Zuscik MJ Hilton MJ Zhang X Chen D O'Keefe RJ. Regulation of chondrogenesis and chondrocyte differentiation by stress. J Clin Invest. (2008) 118(2):429–38. 10.1172/JCI34174 18246193 PMC2214711

[16] Cancel M Grimard G Thuillard-Crisinel D Moldovan F Villemure I. Effects of *in vivo*static compressive loading on aggrecan and type II and X collagens in the rat growth plate extracellular matrix. Bone. (2009) 44(2):306–15. 10.1016/j.bone.2008.09.005 18849019

[17] Stokes IA Spence H Aronsson DD Kilmer N. Mechanical modulation of vertebral body growth. Implications for scoliosis progression. Spine. (1996) 21(10):1162–67. 10.1097/00007632-199605150-00007 8727190

[18] Valteau B Grimard G Londono I Moldovan F Villemure I. *In vivo* dynamic bone growth modulation is less detrimental but as effective as static growth modulation. Bone. (2011) 49(5):996–1004. 10.1016/j.bone.2011.07.008 21784187

[19] Lin H Aubin CE Parent S Villemure I. Mechanobiological bone growth: comparative analysis of two biomechanical modeling approaches. Med Biol Eng Comput. (2009) 47(4):357–66. 10.1007/s11517-008-0425-9 19048322

[20] Villemure I Stokes IA. Growth plate mechanics and mechanobiology. A survey of present understanding. J Biomech. (2009) 42(12):1793–803. 10.1016/j.jbiomech.2009.05.021 19540500 PMC2739053

[21] Zhou H Xu D Quan W Gao Z Xiang L Gu Y. Are there changes in the foot biomechanics during the before and after fifth metatarsal fracture running stance phase? iScience. (2025) 28(5):112432. 10.1016/j.isci.2025.112432 40330882 PMC12051627

[22] Castro-Abril HA Gutiérrez ML DA G-A. Proximal femoral growth plate mechanical behavior: comparison between different developmental stages. Comput Biol Med. (2016) 76:192–201. 10.1016/j.compbiomed.2016.07.011 27479492

[23] Kandzierski G Matuszewski L Wojcik A. Shape of growth plate of proximal femur in children and its significance in the aetiology of slipped capital femoral epiphysis. Int Orthop. (2012) 36(12):2513–20. 10.1007/s00264-012-1699-y 23138967 PMC3508053

[24] Yadav P Fernandez MP Gutierrez-Farewik EM. Influence of loading direction due to physical activity on proximal femoral growth tendency. Med Eng Phys. (2021) 90:83–91. 10.1016/j.medengphy.2021.02.008 33781483

[25] Yadav P Shefelbine SJ Gutierrez-Farewik EM. Effect of growth plate geometry and growth direction on prediction of proximal femoral morphology. J Biomech. (2016) 49(9):1613–19. 10.1016/j.jbiomech.2016.03.039 27063249

[26] Bergmann G Deuretzbacher G Heller M Graichen F Rohlmann A Strauss J. Hip contact forces and gait patterns from routine activities. J Biomech. (2001) 34(7):859–71. 10.1016/s0021-9290(01)00040-9 11410170

[27] Bergmann G Graichen F Rohlmann A. Hip joint loading during walking and running, measured in two patients. J Biomech. (1993) 26(8):969–90. 10.1016/0021-9290(93)90058-M 8349721

[28] Bergmann G Graichen F Rohlmann A Bender A Heinlein B Duda GN. Realistic loads for testing hip implants. Biomed Mater Eng. (2010) 20(2):65–75. 10.3233/BME-2010-0616 20592444

[29] Alexander J Hobbs SJ May K Northrop A Brigden C Selfe J. Postural characteristics of female dressage riders using 3D motion analysis and the effects of an athletic taping technique: a randomised control trial. Phys Ther Sport. (2015) 16(2):154–61. 10.1016/j.ptsp.2014.09.005 25662002

[30] Langerak NG Veerbeek BE Fieggen AG Lamberts RP. Gait status 26–35 years after selective dorsal rhizotomy: a 9 year follow up study. Gait Posture. (2022) 91:284–89. 10.1016/j.gaitpost.2021.10.031 34798419

[31] Martini E Boldo M Aldegheri S Valè N Filippetti M Smania N. Enabling gait analysis in the telemedicine practice through portable and accurate 3D human pose estimation. Comput Methods Programs Biomed. (2022) 225:107016. 10.1016/j.cmpb.2022.107016 35907374

[32] Carter CW Campbell A Whitney D Feder O Kingery M Baron S. Characterizing cam-type hip impingement in professional women's Ice hockey players. Phys Sportsmed. (2021) 49(2):203–06. 10.1080/00913847.2020.1808434 32799611

[33] Xu J Zhan S Ling M Jiang D Hu H Sheng J. Biomechanical analysis of fibular graft techniques for nontraumatic osteonecrosis of the femoral head: a finite element analysis. J Orthop Res. (2020) 15(1):1–10. 10.1186/s13018-020-01867-4 PMC7433362 32807218

[34] Todd C Karlsson J Baranto A. Resolving anterior hip pain in a young male footballer following arthroscopic surgery for femoroacetabular impingement syndrome: a case report. J Bodyw Mov Ther. (2020) 24(1):63–8. 10.1016/j.jbmt.2019.05.027 31987564

[35] Sadeghian SM Lewis CL Shefelbine SJ. Predicting growth plate orientation with altered hip loading: potential cause of cam morphology. Biomech Model Mechan. (2020) 19:701–12. 10.1007/s10237-019-01241-2 31712938

[36] Kainz H Killen BA Wesseling M Perez-Boerema F Pitto L Garcia Aznar JM. A multi-scale modelling framework combining musculoskeletal rigid-body simulations with adaptive finite element analyses, to evaluate the impact of femoral geometry on hip joint contact forces and femoral bone growth.PLoS One. (2020) 15(7):e0235966. 10.1371/journal.pone.0235966 32702015 PMC7377390

[37] Mansournia MA Collins GS Nielsen RO Nazemipour M Jewell NP Altman DG. A checklist for statistical assessment of medical papers (the CHAMP statement): explanation and elaboration. Br J Sports Med. (2021) 55(18):1009–17. 10.1136/bjsports-2020-103652 PMC9110112 33514558

[38] Agricola R Heijboer MP Ginai AZ Roels P Zadpoor AA Verhaar JAN. A cam deformity is gradually acquired during skeletal maturation in adolescent and young male soccer players: a prospective study with minimum 2-year follow-up. Am J Sports Med. (2014) 42(4):798–806. 10.1177/0363546514524364 24585362

[39] Siebenrock KA Behning A Mamisch TC Schwab JM. Growth plate alteration precedes cam-type deformity in elite basketball players. Clin Orthop Relat R. (2013) 471(4):1084–91. 10.1007/s11999-012-2740-6 PMC3585998 23247816

[40] Albers CE Steppacher SD Haefeli PC Werlen S Hanke MS Siebenrock KA. Twelve percent of hips with a primary cam deformity exhibit a slip-like morphology resembling sequelae of slipped capital femoral epiphysis. Clin Orthop Relat R. (2015) 473(4):1212–23. 10.1007/s11999-014-4068-x PMC4353527 25448326

[41] Gao J Williams JL Roan E. Multiscale modeling of growth plate cartilage mechanobiology. Biomech Model Mechan. (2017) 16(2):667–79. 10.1007/s10237-016-0844-8 27770213

[42] Wang N Zhang X Rothrauff B Fritch MR Chang A Yeung M. Novel role of estrogen receptor-α on regulating chondrocyte phenotype and response to mechanical loading. Osteoarthritis Cartilage. (2022) 30(2):302–14. 10.1016/j.joca.2021.11.002 34767957

[43] Lee W Nims RJ Savadipour A Zhang Q Leddy HA Liu F. Inflammatory signaling sensitizes Piezo1 mechanotransduction in articular chondrocytes as a pathogenic feed-forward mechanism in osteoarthritis. Proc Natl Acad Sci. (2021) 118(13):e2001611118. 10.1073/pnas.2001611118 33758095 PMC8020656

[44] Carter DR Wong M. The role of mechanical loading histories in the development of diarthrodial joints. J Orthop Res. (1988) 6(6):804–16. 10.1002/jor.1100060604 3171761

[45] Carter DR Wong M. Modelling cartilage mechanobiology. Philos Trans R Soc Lond B Biol Sci. (2003) 358(1437):1461–71. 10.1098/rstb.2003.1346 14561337 PMC1693248

[46] Zhang AL. CORR Insights(R): a cam morphology develops in the early phase of the final growth spurt in adolescent ice hockey players: results of a prospective MRI-based study. Clin Orthop Relat R. (2021) 479(5):919–21. 10.1097/CORR.0000000000001648 PMC8052017 33497064

[47] Siebenrock KA Behning A Mamisch CT Schwab JM. Growth plate alteration precedes cam-type deformity in elite basketball players. Clin Orthop Relat R. (2013) 471(4):1084–91. 10.1007/s11999-012-2740-6 PMC3585998 23247816

[48] Polat G Arzu U Dinç E Bayraktar B. Prevalence of femoroacetabular impingement and effect of training frequency on aetiology in paediatric football players. Hip Int. (2019) 29(2):204–08. 10.1177/1120700018781939 29932009

[49] Fernquest S Palmer A Gimpel M Birchall R Broomfield J Wedatilake T. A longitudinal cohort study of adolescent elite footballers and controls investigating the development of cam morphology. Sci Rep. (2021) 11(1):18567. 10.1038/s41598-021-97957-2 34535729 PMC8448877

[50] Hanke MS Schmaranzer F Steppacher SD Reichenbach S Werlen SF Siebenrock KA. A cam morphology develops in the early phase of the final growth spurt in adolescent ice hockey players: results of a prospective MRI-based study. Clin Orthop Relat R. (2021) 479(5):906–18. 10.1097/CORR.0000000000001603 PMC8052031 33417423

[51] Zhang AL. CORR Insights®: a cam morphology develops in the early phase of the final growth spurt in adolescent ice hockey players: results of a prospective MRI-based study. Clin Orthop Relat R. (2021) 479(5):919–21. 10.1097/CORR.0000000000001648 PMC8052017 33497064

[52] Roels P Agricola R Oei EH Weinans H Campoli G Zadpoor AA. Mechanical factors explain development of cam-type deformity. Osteoarthritis Cartilage. (2014) 22(12):2074–82. 10.1016/j.joca.2014.09.011 25241242

[53] Gao J Roan E Williams JL. Regional variations in growth plate chondrocyte deformation as predicted by three-dimensional multi-scale simulations.PLoS One. (2015) 10(4):e0124862. 10.1371/journal.pone.0124862 25885547 PMC4401775

[54] Yadav P Shefelbine SJ Pontén E Gutierrez-Farewik EM. Influence of muscle groups' Activation on proximal femoral growth tendency. Biomech Model Mechan. (2017) 16(6):1869–83. 10.1007/s10237-017-0925-3 PMC5671539 28639152

[55] Morris WZ Napora JK Conry KT Liu RW. Capital femoral epiphyseal extension may confer physeal stability in slipped capital femoral epiphysis. J Pediatr Orthoped. (2019) 39(3):119–24. 10.1097/BPO.0000000000000881 30730415

