# Supplementary material for: Finite element analysis of the proximal femoral growth plate biomechanics: insights into cam deformity risk of adolescent athletes
Source: Front Sports Act Living. 2026 Jul 14;8:1746084. doi: 10.3389/fspor.2026.1746084 (PMC13422550; doi:10.3389/fspor.2026.1746084)
Supplement: Supplementary file 1 [file Datasheet1.docx]

**Supplementary Materials**


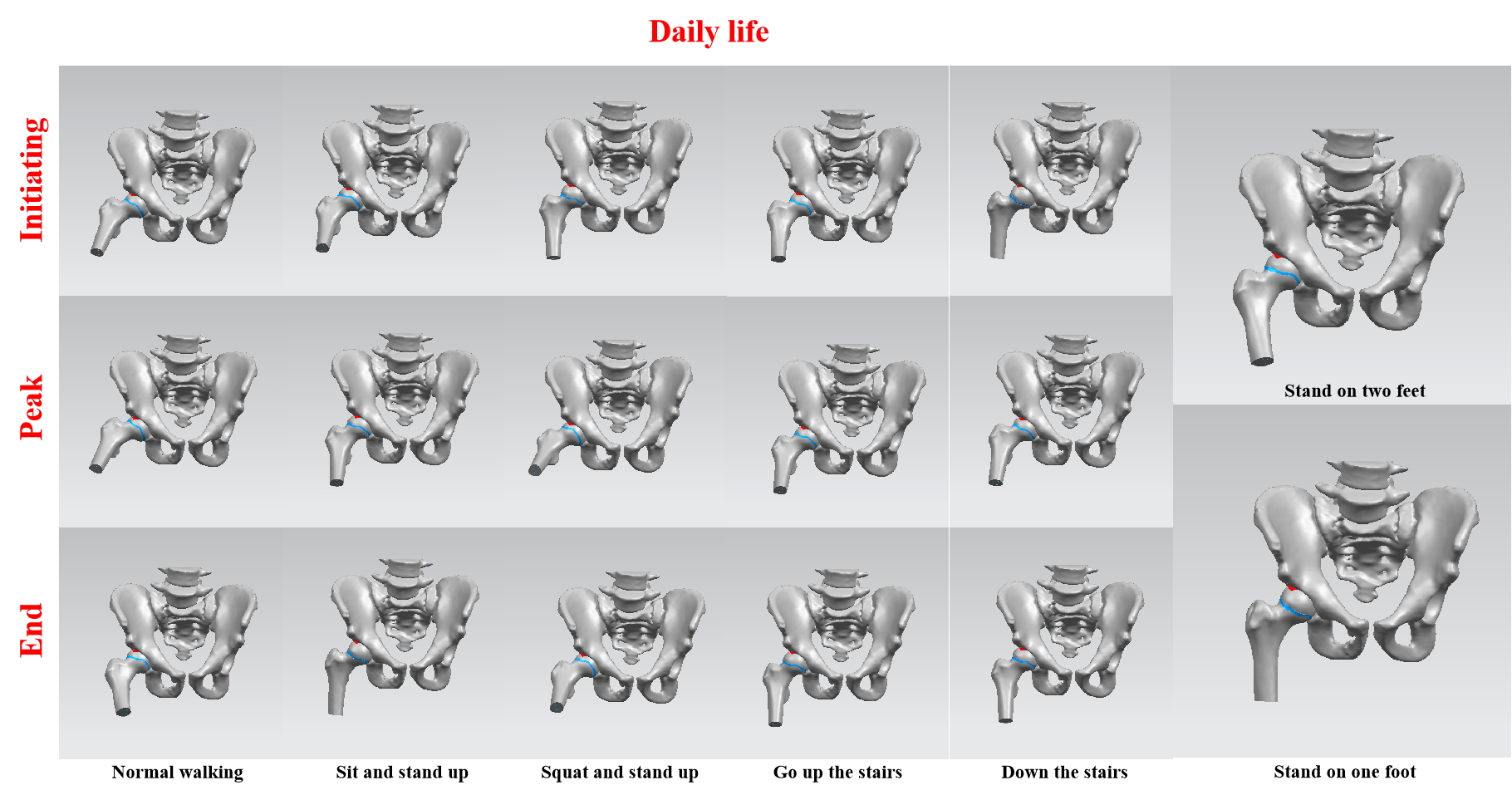


**Figure S1**. Hip joint position under mechanical loading in daily life.


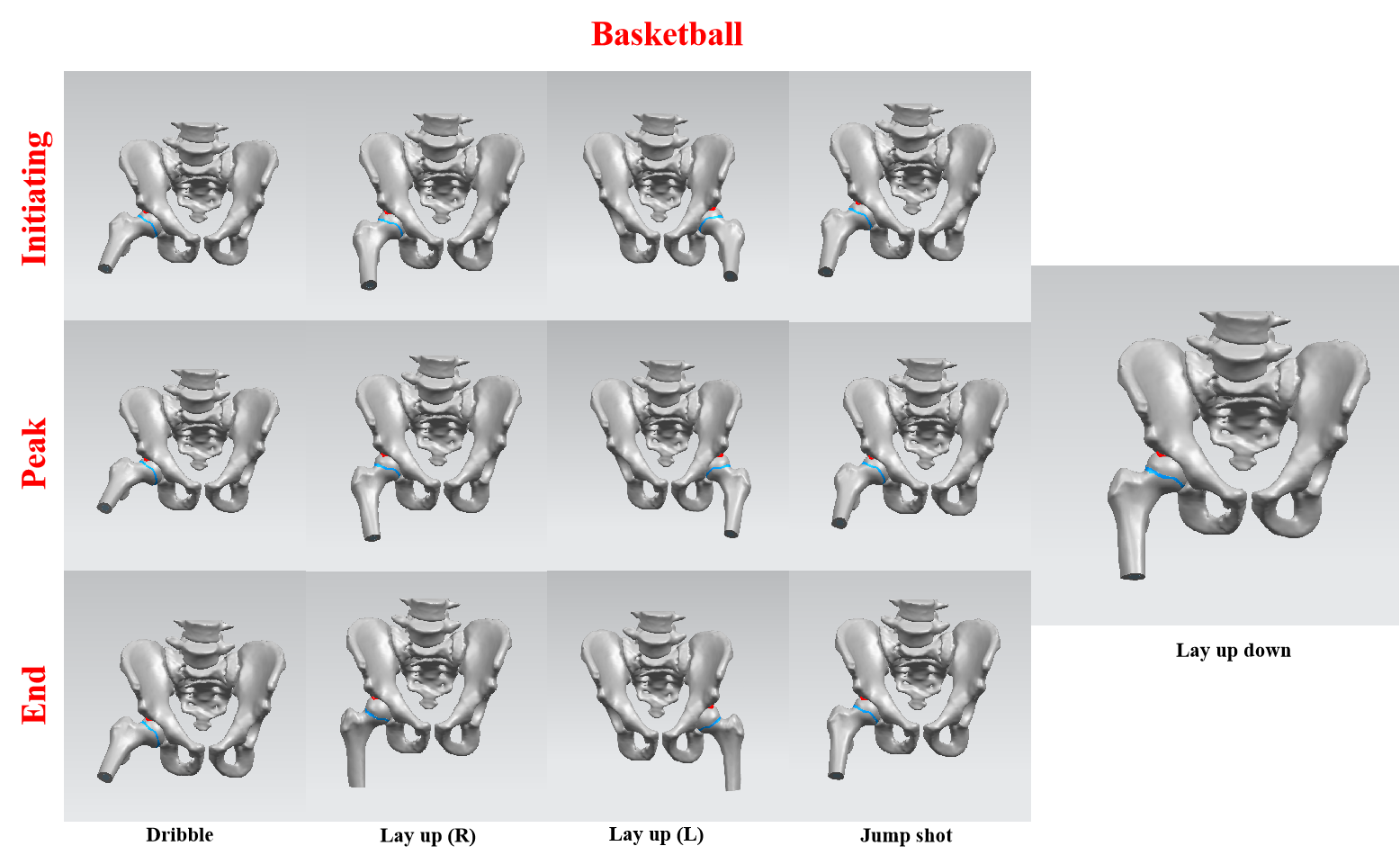


**Figure S2**. Hip joint position under mechanical loading in basketball.


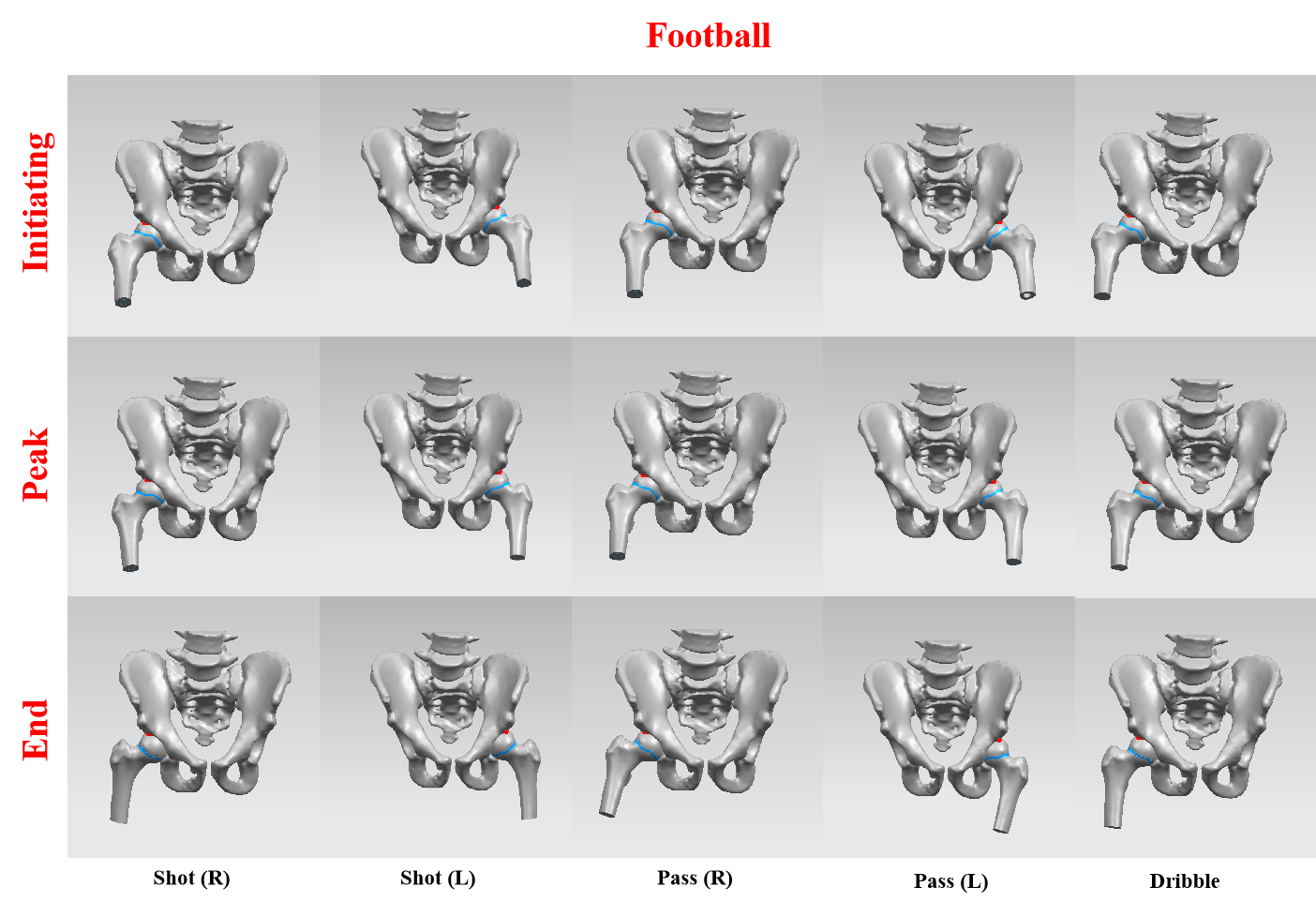


**Figure S3**. Hip joint position under mechanical loading in football.


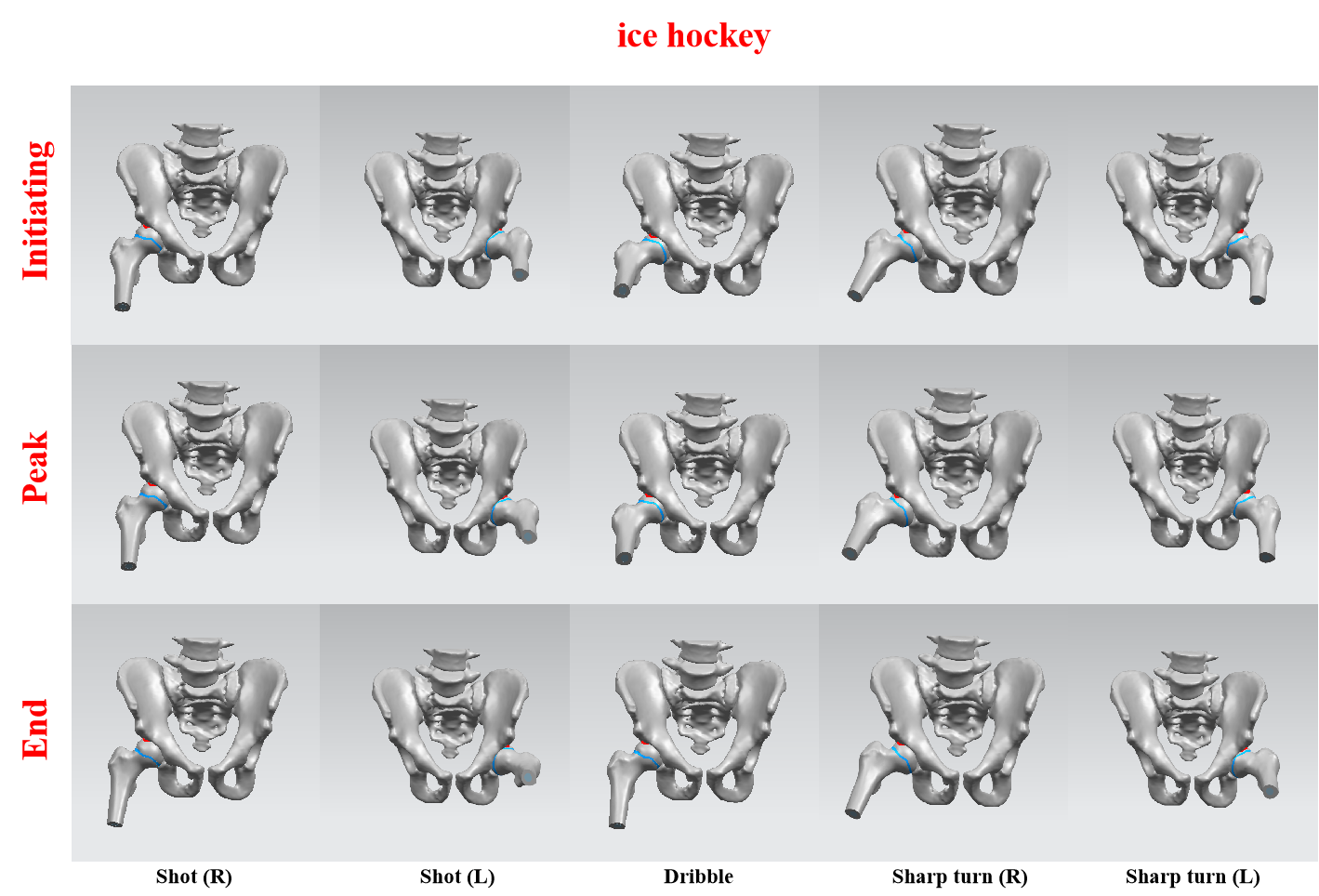


**Figure S4**. Hip joint position under mechanical loading in ice hockey.


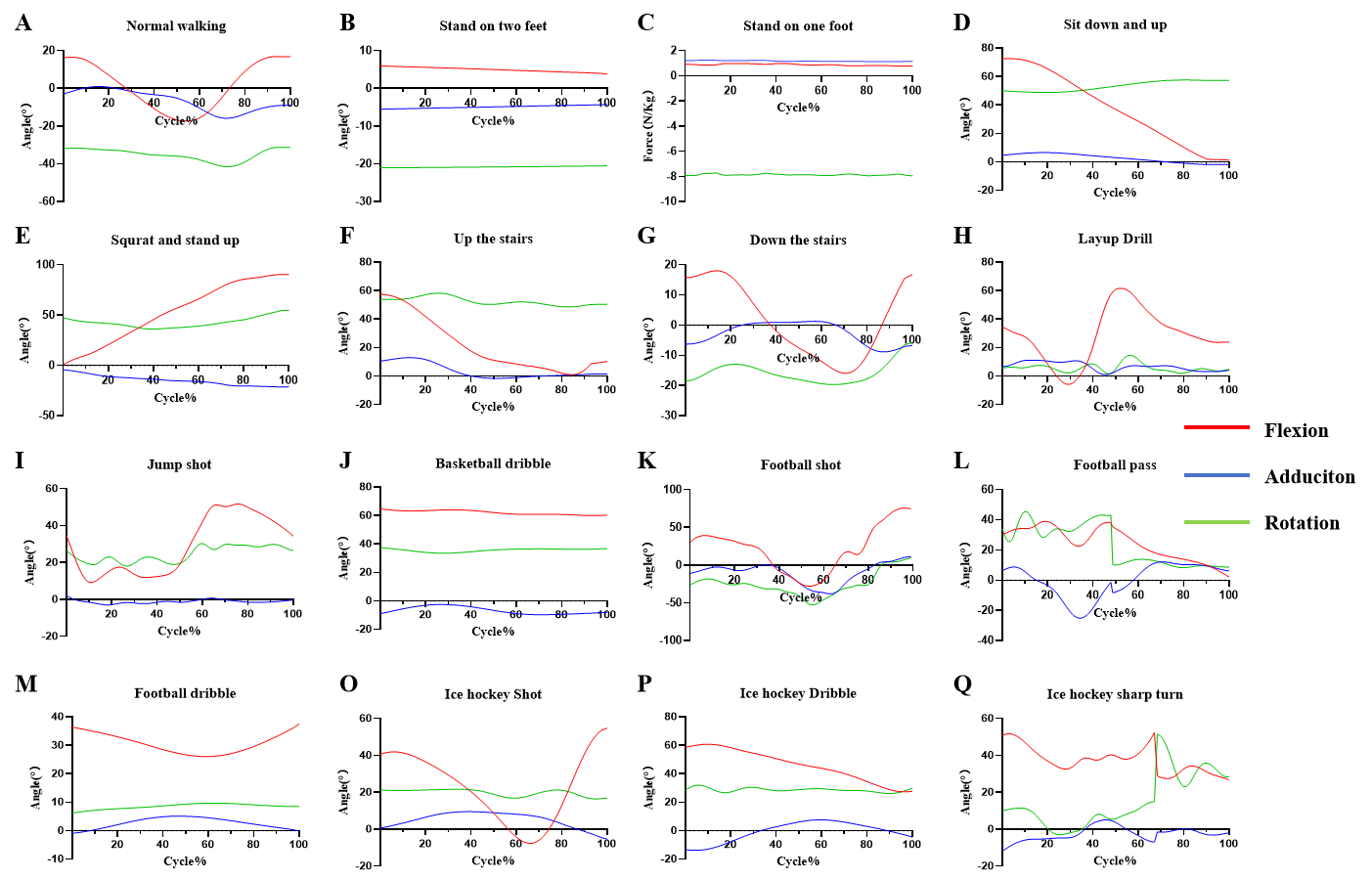


**Figure S5**. Hip joint position under mechanical loading in in different activities (one cycle).


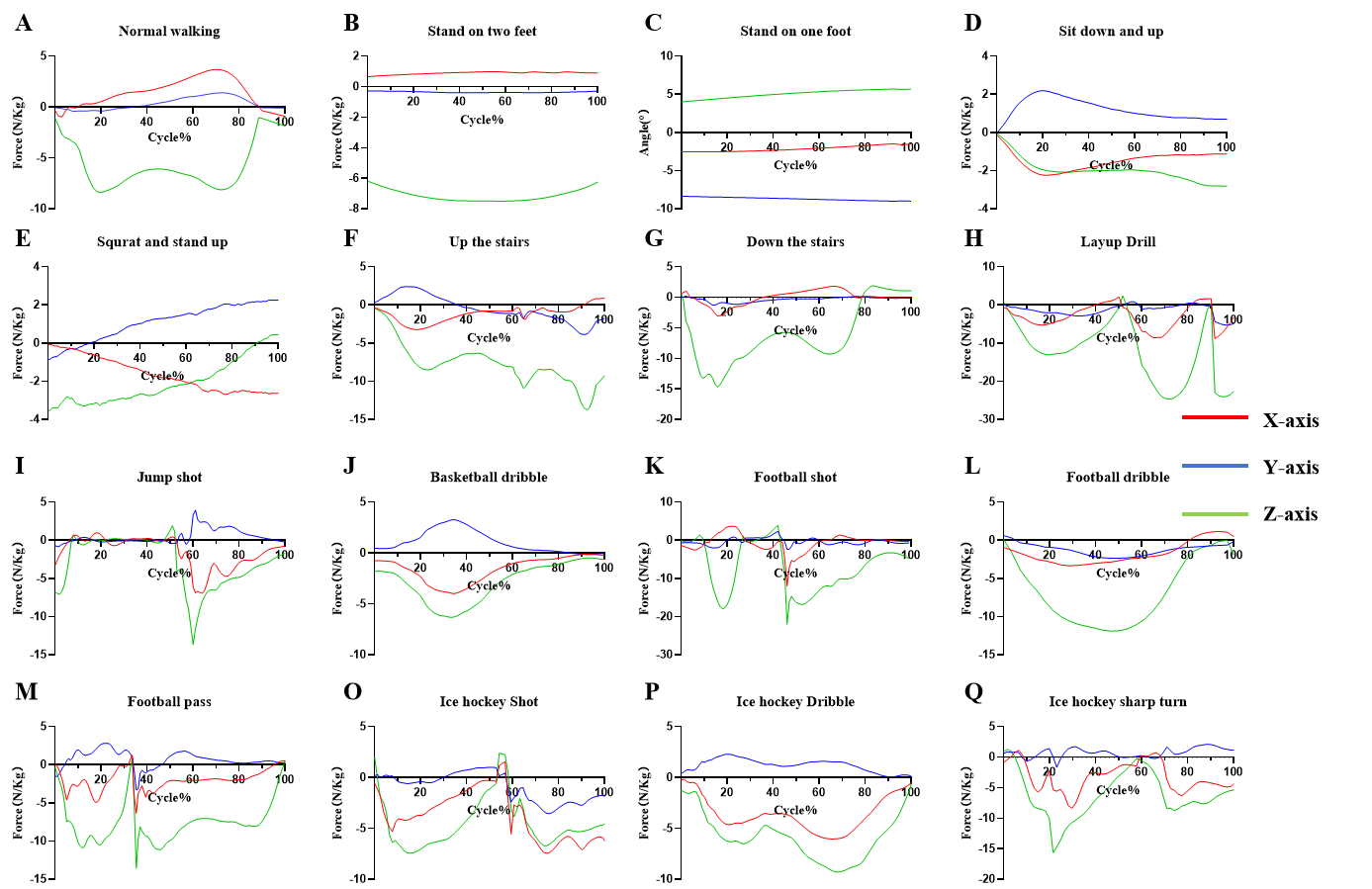


**Figure S6**. Hip joint angle under mechanical loading in in different activities (one cycle).

**Table S1.** Mean values of hip force and hip angle under mechanical loading in different activities.

| Classification | Status | Hip Angle (°) | | | Hip Force (N) | | |
| --- | --- | --- | --- | --- | --- | --- | --- |
|  |  | X | Y | Z | x | y | z |
| Normal walking | 1 | 37.974 | -9.993 | 22.537 | -37.124 | 19.179 | -71.123 |
|  | 2 | 42.660 | -16.106 | 18.036 | 121.669 | -64.764 | -511.246 |
|  | 3 | 38.228 | 1.723 | -1.921 | -2.804 | -0.858 | -99.112 |
| Stand on two feet | 1 | 24.763 | 2.347 | -4.342 | 17.072 | -13.933 | -184.825 |
| Stand on one foot | 1 | -3.356 | -2.012 | -12.441 | 19.355 | -47.180 | -475.058 |
| Sit and stand up | 1 | 41.836 | 4.441 | 21.252 | 4.575 | -17.147 | -191.104 |
|  | 2 | 31.349 | 4.579 | 18.160 | -136.222 | 28.109 | -155.677 |
|  | 3 | -1.373 | -5.831 | 6.491 | -111.191 | 24.818 | -64.225 |
| Squat and stand up | 1 | 16.232 | 1.386 | 13.958 | -1.670 | -13.650 | -194.685 |
|  | 2 | 55.813 | 2.536 | 27.299 | -157.556 | 42.557 | -141.934 |
|  | 3 | 49.261 | 1.927 | 17.010 | -208.227 | 83.390 | -10.146 |
| Go up stairs | 1 | 30.474 | -2.386 | 5.615 | -63.226 | 19.867 | -70.664 |
|  | 2 | 36.429 | 2.142 | 17.567 | -187.232 | 15.498 | -399.501 |
|  | 3 | 21.101 | 5.169 | 24.083 | -37.004 | -44.763 | -417.723 |
| Down stairs | 1 | -5.483 | -7.030 | 4.210 | 18.535 | -15.693 | -120.988 |
|  | 2 | 29.501 | -5.798 | 12.359 | -90.815 | -0.309 | -836.069 |
|  | 3 | 19.029 | 3.638 | 16.537 | 8.676 | -20.436 | -299.311 |
| Basketball Dribble | 1 | 41.34 | -15.52 | 17.07 | -23.68 | 8.28 | -75.23 |
|  | 2 | 49.24 | -18.25 | 16.41 | -255.09 | 72.83 | -446.85 |
|  | 3 | 42.06 | -21.33 | 12.13 | -30.71 | -4.57 | -72.47 |
| Layup Drill(R) | 2 | 24.66 | 3.50 | -1.79 | -174.12 | -5.95 | -851.57 |
| Layup Drill(L) | 2 | 29.29 | 7.91 | 6.20 | -353.45 | -6.54 | -1194.35 |
| Layup Down | 1 | 17.04 | -0.75 | 2.42 | -601.88 | -38.49 | -1754.05 |
| Jump Shot | 1 | 39.69 | -5.44 | 14.98 | -162.58 | 25.62 | -190.98 |
|  | 2 | 44.63 | -3.81 | 15.49 | -227.49 | -16.74 | -380.45 |
|  | 3 | 29.56 | -4.21 | 11.26 | -245.46 | 37.71 | -446.69 |
| Football Shot（R） | 2 | 23.37 | 6.32 | 13.31 | -151.67 | -26.94 | -846.10 |
| Football Shot（L） | 2 | 20.86 | 1.20 | 9.75 | -261.97 | -15.14 | -938.72 |
| Football Pass（R） | 2 | 30.46 | -1.68 | 11.10 | -180.94 | 89.98 | -746.95 |
| Football Pass（L） | 2 | 25.35 | -4.74 | -1.45 | -205.31 | -95.74 | -555.79 |
| Football Dribble | 1 | 28.80 | -5.22 | 6.91 | -53.74 | 9.44 | -88.03 |
|  | 2 | 21.12 | -4.45 | 5.64 | -162.65 | 9.71 | -667.28 |
|  | 3 | 6.76 | -6.58 | 3.76 | -11.50 | -37.96 | -108.88 |
| Ice hockey Shoot(R) | 2 | 31.07 | 5.99 | 20.49 | -182.97 | -30.45 | -582.16 |
| Ice hockey Shoot(L) | 3 | 77.72 | 6.74 | 7.51 | -370.67 | -58.22 | -268.53 |
| Ice hockey Dribble | 1 | 56.97 | -1.47 | 12.13 | -56.50 | 26.26 | -55.88 |
|  | 2 | 51.62 | 3.90 | 10.78 | -324.06 | -10.04 | -429.82 |
|  | 3 | 23.79 | -4.42 | 11.18 | -50.40 | 61.09 | -137.39 |
| Ice hockey Sharp turn（R） | 2 | 47.35 | -21.78 | 14.28 | -241.79 | 95.01 | -380.08 |
| Ice hockey Sharp turn（L） | 2 | 41.70 | -8.27 | -4.05 | -282.13 | -63.64 | -580.19 |

**Hip Angle** X represents the flexion and extension angle of the hip, with positive values during flexion and negative values during extension; Y represents the adduction and abduction angle, with positive values for adduction and negative values for abduction; Z represents the external and internal rotation angle, with positive values for external rotation and negative values for internal rotation.

**Hip Force** x represents the force in the forward-backward direction, with positive values for forces directed forward and negative values for forces directed backward; y represents the force in the horizontal direction, with positive values for forces directed to the left and negative values for forces directed to the right; z represents the force in the vertical direction, with positive values for forces directed upward and negative values for forces directed downward.

**Table S2**. Mesh parameters for each anatomical component

| **Component** | **Element type** | **Mesh size** | **Element count** | **Node count** |
| --- | --- | --- | --- | --- |
| Growth Plate | SOLID187 (10-node tetrahedron) | 0.5 mm | 443110 | 631157 |
| Metaphysis/Cancellous Bone | SOLID187 | 1.0 mm | 122714 | 174536 |
| Diaphysis/Cancellous Bone | SOLID187 | 1.0 mm | 601518 | 8344606 |
| Metaphysis/Cortical Bone | SOLID187 | 1.0 mm | 74843 | 116759 |
| Diaphysis/Cortical Bone | SOLID187 | 1.0 mm | 400305 | 596378 |
